# Federated clustered multi-domain learning for health monitoring

**DOI:** 10.1038/s41598-024-51344-9

**Published:** 2024-01-09

**Authors:** Shiyi Jiang, Yuan Li, Farshad Firouzi, Krishnendu Chakrabarty

**Affiliations:** 1 Department of Electrical and Computer Engineering, Duke University, Durham, NC 27708 USA; 2 Division of Natural and Applied Sciences, Duke Kunshan University, Kunshan, 215316 Jiangsu China; 3 School of Electrical, Computer and Energy Engineering, Arizona State University, Tempe, AZ 85281 USA

**Keywords:** Health care, Engineering

## Abstract

Wearable Internet of Things (WIoT) and Artificial Intelligence (AI) are rapidly emerging technologies for healthcare. These technologies enable seamless data collection and precise analysis toward fast, resource-abundant, and personalized patient care. However, conventional machine learning workflow requires data to be transferred to the remote cloud server, which leads to significant privacy concerns. To tackle this problem, researchers have proposed federated learning, where end-point users collaboratively learn a shared model without sharing local data. However, data heterogeneity, i.e., variations in data distributions within a client (intra-client) or across clients (inter-client), degrades the performance of federated learning. Existing state-of-the-art methods mainly consider inter-client data heterogeneity, whereas intra-client variations have not received much attention. To address intra-client variations in federated learning, we propose a federated clustered multi-domain learning algorithm based on ClusterGAN, multi-domain learning, and graph neural networks. We applied the proposed algorithm to a case study on stress-level prediction, and our proposed algorithm outperforms two state-of-the-art methods by 4.4% in accuracy and 0.06 in the F1 score. In addition, we demonstrate the effectiveness of the proposed algorithm by investigating variants of its different modules.

## Introduction

Wearable Internet of Things (WIoT) and Artificial Intelligence (AI) technology have revolutionized the healthcare sector in recent years^1^. WIoT devices provide seamless data collection, and AI techniques allow automated data analysis, expediting medical diagnosis and treatments, thus improving the quality of patient care^2^. Integration of the edge and cloud computing with healthcare enables remote health monitoring as a complement to the physician-centered healthcare model^3^. Although WIoT and AI technology significantly improved healthcare data collection and analysis, existing solutions are confronted with privacy concerns^4^. Healthcare data is highly sensitive and the process of accessing and analyzing the data is rigorously regulated by policies and regulations, such as the Health Insurance Portability and Accountability Act (HIPAA)^5^. Existing work involves data transmission to the remote cloud server for centralized machine learning, which poses high risks of private information leakage^6^. Privacy-Preserving Machine Learning (PPML) has thus emerged to address this problem. Within PPML, researchers have proposed to combine Machine Learning (ML) with techniques such as homomorphic encryption^7^, secure multi-party computation^8^, differential privacy^9^, and, particularly, Federated Learning (FL) to address privacy concerns.

FL enables collaborative learning of a shared model across multiple decentralized edge devices or servers (i.e., clients), holding local privacy-sensitive data samples without exchanging them^10–12^. The deployment of FL to healthcare promises to allow collaboration between institutions without exchanging sensitive data^13,14^. For instance, Lee et al. learned patient similarity across hospitals via FL^15^. Brisimi et al. created a prediction model for hospitalization due to cardiac diseases under the FL setting^16^. Huang et al. developed a community-based federated machine learning algorithm to predict mortality and ICU stay time using electronic medical records^17^.

### Limitations of previous work

**Figure 1:**
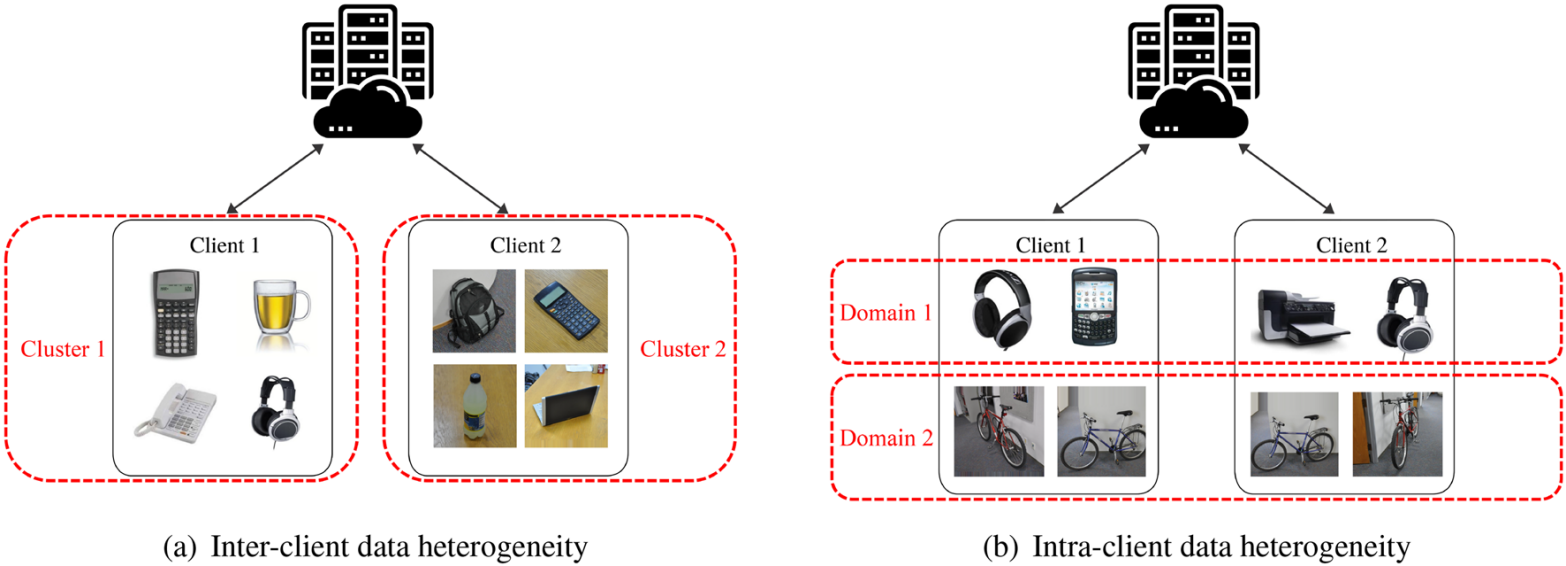
Illustration of inter-client and intra-client data heterogeneity (object images adapted from the Office dataset^23^).

Conventional FL algorithms assume that local data from each client is independent and identically distributed (i.i.d)^18^. However, in practice, a dataset is typically composed of different domains (i.e., data distributions), which are non-independent and not identically distributed (non-i.i.d). For instance, different styles and backgrounds of the images and various languages used in texts are considered distinct domains. Such domain difference, i.e., data heterogeneity, adversely impacts the generalization ability of the global FL model across all data, resulting in degradation in task performance^19^. Data heterogeneity occurs in the following two scenarios in the FL scheme:

- Inter-client data heterogeneity: It refers to the case when there exist domain differences across clients. For example, as illustrated in Fig. 1a, images from different clients have diverse backgrounds, viewpoints, and image resolutions, indicating that they belong to different domains. Inter-client data heterogeneity deteriorates the performance of the conventional one-size-fits-all FL algorithms such as FedAvg^10^ since the aggregated models obscure individual differences.
- Intra-client data heterogeneity: It refers to the case when there exist domain differences within a client. As illustrated in Fig. 1b, data from a single client belongs to multiple domains, having different data distributions. Intra-client data heterogeneity is a common issue in the field of healthcare. Long et al.^20^ investigated cardiorespiratory activities and electrophysiological signals at different stages of sleep and found that the same patient’s body signals vary by stage. In addition, Karimian et al.^21^ indicate that the impact of the intra-client variation of electrocardiogram (ECG) signals cannot be neglected. The aforementioned examples illustrate how a single individual/client can encompass multiple data distributions (domains), stemming from the different patterns involved in a physiological event over time. Note that patterns of the physiological attributes (domains) for a disease of interest over time are usually unknown ahead of time^22^.

There exists literature on alleviating inter-client data heterogeneity in FL. For example, Ghosh et al.^24^ and Briggs et al.^25^ proposed to divide clients into clusters according to their underlying similarities and train a model for each cluster. Fallah et al.^26^ suggested that Model-Agnostic Meta-Learning (MAML)^27^ is a promising framework for building a personalized model for each client. Smith et al.^28^ integrated multi-task learning into FL, which considers each client having a model for a different task and trains those correlated models simultaneously^29^. However, previous approaches on building customized models for a subset/an individual client fail to capture the data heterogeneity within each client.

Intra-client heterogeneity has not been studied much, and there is only a limited body of literature addressing this issue. In this context, Caldarola et al.^30^ proposed to model data heterogeneity by identifying domains for each client via a combination of knowledge distillation, domain-specific learning, and graph representation learning techniques. Shenaj et al.^31^ further extended the idea of learning across domains to tackle data heterogeneity via style transfer and server-side pre-training. However, their assumptions include prior knowledge of the domains and their relations, which is not applicable to our context. Consequently, we develop an algorithm that addresses intra-client data heterogeneity with unknown domains in FL.

### Motivation and paper contributions

Our work is inspired by multi-domain learning. Multi-domain learning learns correlated domains simultaneously by training a set of models and then adapting them to specific domains^32^. There are two mainstream categories of methods for multi-domain learning. One set of approaches utilizes adaptors, which are small neural network modules attached to a large pre-trained neural network model^33,34^. The pre-trained portion of the model learns shared information across domains (i.e., domain-agnostic), and the model obtains domain-specific knowledge by training the adaptors. The other set of approaches utilizes model parameter sharing. Studies^35,36^ show that retraining later layers of the neural network models can effectively capture domain-specific information.

There is only a limited amount of prior work on incorporating multi-domain learning into the context of FL. Parekh et al.^37^ performed FL in the multi-domain, multi-task setting for medical image object detection and segmentation. Li et al.^38^ presented FedH2L, which utilizes mutual learning to tackle inter-client data and model architecture heterogeneity. Sun et al.^39^ utilized partial model parameter sharing of the global model to mitigate inter-client data heterogeneity due to the cross-domain effect. Elvebakken et al.^40^ suggested introducing adaptors in the federated learning system to reduce communication overhead. However, none of the existing federated multi-domain learning literature addresses the problem of intra-client data heterogeneity in FL.

We thus propose to integrate multi-domain learning into FL to identify domain differences within individual clients. We perform federated clustering to learn domain categories within each client. We then construct a neural network architecture shared across all domains. We enable parameters at selected layers of the client model to be domain-specific and the rest of the parameters to be domain-agnostic. In addition, we introduce an auxiliary graph neural network to fine-tune the domain-agnostic and domain-specific knowledge. The key contributions of this paper are threefold:

- We identify a crucial source of data heterogeneity, i.e., within-client data heterogeneity, and propose a novel federated clustered multi-domain learning algorithm to overcome this problem.
- We employ a graph attention network as an auxiliary representation to connect and capture the implicit relationships between different domains.
- We validate the effectiveness of the proposed algorithm using a case study on stress-level prediction. The proposed method improves the overall accuracy and the F1 score by over 4.41% and over 7.8%, respectively, compared to two state-of-the-art methods. The performance of our model is also robust for all individual clients.

The organization of the rest of the paper is as follows: We introduce the proposed method in the Method section. In the Results section, we specify the dataset and experimental setup used for the case study; we demonstrate the effectiveness of the proposed algorithm by comparing it with state-of-the-art approaches and analyzing different modules of the proposed method. Finally, we conclude the paper in the Conclusion section.

## Method

**Figure 2:**
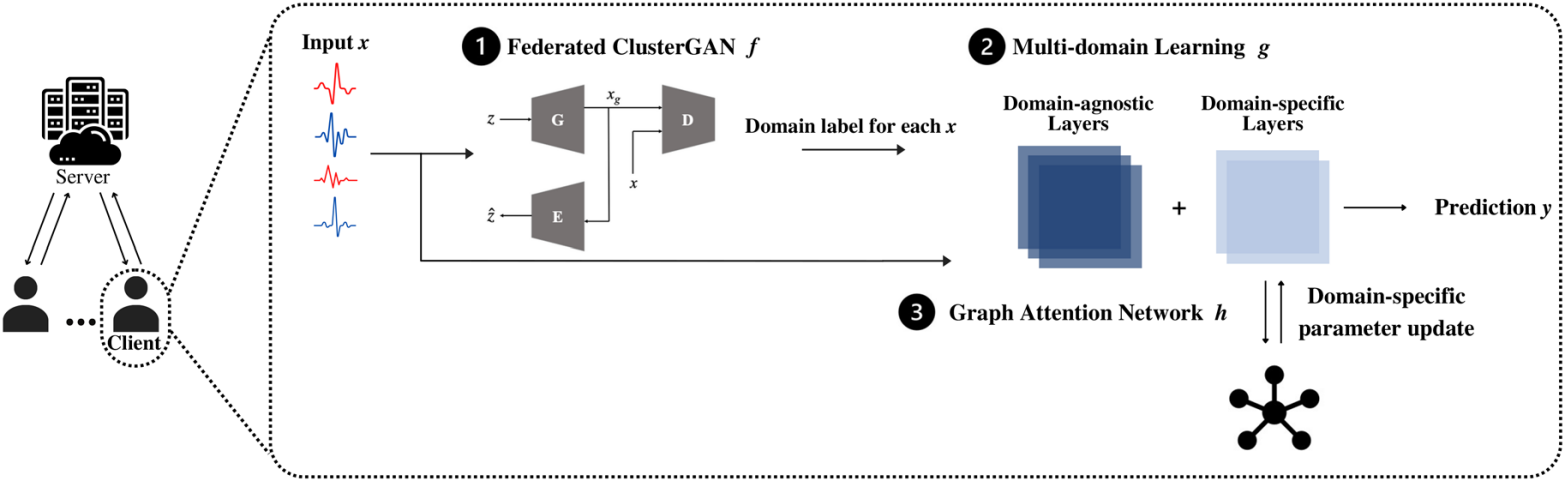
Overall framework of the proposed federated clustered multi-domain learning algorithm.

As explained in the Introduction, existing literature alleviates only inter-client data heterogeneity; it fails to capture intra-client data heterogeneity. This section presents a clustered multi-domain FL algorithm to mitigate the ill effects of intra-client data heterogeneity. As depicted in Fig. 2, the proposed algorithm comprises three modules: a federated clustering module, a multi-domain learning module, and a graph-based domain fine-tuning module. The federated clustering module assigns domain labels to data samples at each client in a shared domain space. The multi-domain learning module enables the learning of domain-specific knowledge across all clients. A graph attention network is utilized at each client to facilitate fine-tuning of the domain-agnostic and domain-specific knowledge in the graph-based domain fine-tuning module.

Specifically, given a dataset $D = \{(X_i, Y_i), i=1, \dots , N\}$ where $X_i$ is the set of images from Client *i*, $Y_i$ is the set of corresponding labels for $X_i$, and *N* is the number of clients, the objective of the proposed algorithm is to learn a function $F(w; \theta ; \phi ; \omega ) = \{f(w), g(\theta ; \phi ), h(\omega )\}: X_i \rightarrow Y_i$. The federated clustering module $f(w): X_i \rightarrow \mathscr {D}_i = \{d, d \in \{1, \dots , K\}\}$ assigns domain labels $\mathscr {D}_i$ to images $X_i$, where *w* is the module parameter, *d* is the domain label for a single image sample in $X_i$, and *K* is the number of domains obtained from the estimation algorithm from our prior work^41^. The multi-domain learning module $g(\theta ; \phi ): X_i^d \rightarrow Y_i$ maps the data labeled with domain tags $X_i^d$ to the set of labels $Y_i$, where $\theta$ is the domain-agnostic parameter and $\phi$ is the domain-specific parameter. In the last module, we fine-tune parameter $\phi$ by applying a graph attention network $h(\omega ): \phi \rightarrow \phi '$. We update $g(\theta ; \phi ')$ with the fine-tuned $\phi '$ and obtain the final classification output $Y_i$. We next explain each module in detail.

### Federated ClusterGAN

**Figure 3:**
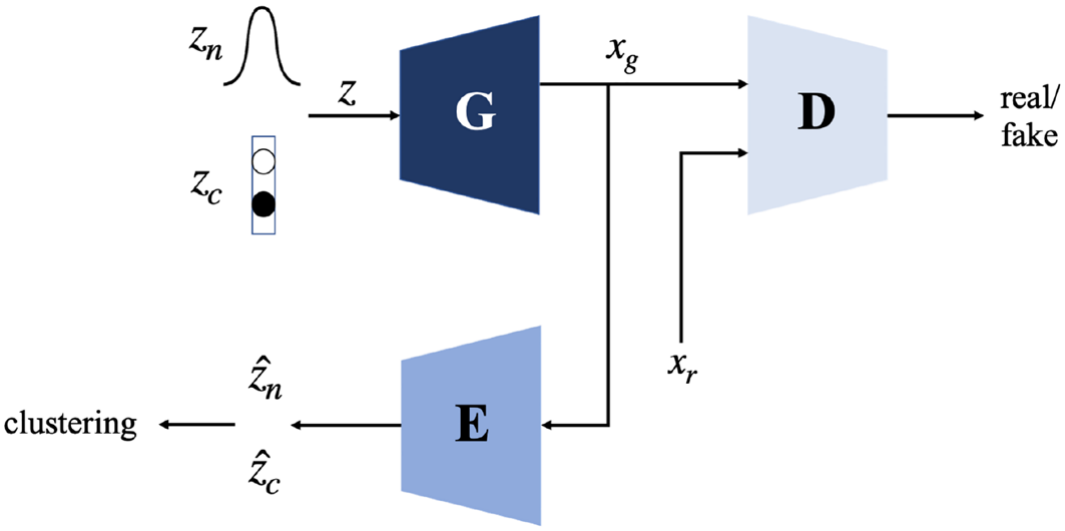
ClusterGAN architecture.

The federated ClusterGAN groups each set of $X_i$ into *K* domains, where these domains are shared across all clients. It is known that clustering in the latent space utilizing ClusterGAN provides more stable clustering results compared to clustering in the data space^42^. Unlike conventional Generative Adversarial Networks, ClusterGAN is composed of three deep neural networks: a generator $\textbf{G}$, a discriminator $\textbf{D}$, and an encoder $\textbf{E}$, as shown in Fig.3 parameterized by $\Theta _{\textbf{G}}$, $\Theta _{\textbf{D}}$, and $\Theta _{\textbf{E}}$, respectively.

The generator $\textbf{G}$ maps the latent space to the data space. It samples from the latent space *z*, which consists of a continuous normal distribution $z_n$ and a discrete one-hot vector $z_c$, and generates artificial sample $x_{g}$. Formally, $z = (z_n, z_c), \; z_n \sim N(0, \sigma ^2I), \; z_c = e_k, \; k \in \{1, \dots , K\}$, where $e_k$ is the $k^{th}$ one-hot vector, and *K* is the number of clusters. The discriminator $\textbf{D}$ projects the artificial sample $x_{g}$ and the real sample $x_{r}$ in the data space to a real value that indicates the probability of the sample being real. The encoder $\textbf{E}$ generates the discriminative latent space variables $\hat{z_n}$ and $\hat{z_c})$ using $x_{g}$. The objective of ClusterGAN training is to minimize the loss function presented in Eq. (1)^42^, where $\mathbb {P}_x^r$ is the distribution of real data samples, $\mathbb {P}_z$ is the prior distribution in the latent space. $q(\cdot )$ is the quality function which is $\log (x)$ for conventional GAN and *x* for Wasserstein GAN. $\mathscr {L}(\cdot )$ is the cross-entropy loss, and $\beta _n$ and $\beta _c$ are regularization coefficients.

1 $$\begin{aligned} {\begin{matrix} \min _{\Theta _{\textbf{G}}, \Theta _{\textbf{E}}} \max _{\Theta _{\textbf{D}}}&\mathop {\mathbb {E}}_{x \sim \mathbb {P}_x^r} q (\textbf{D}(x)) + \mathop {\mathbb {E}}_{z \sim \mathbb {P}_z} q (1 - \textbf{D}(\textbf{G}(z))) + \beta _n \mathop {\mathbb {E}}_{z \sim \mathbb {P}_z} ||z_n - \textbf{E}(\textbf{G}(z_n))||_2^2 + \beta _c \mathop {\mathbb {E}}_{z \sim \mathbb {P}_z} \mathscr {L}(z_c, \textbf{E}(\textbf{G}(z_c))) \end{matrix}} \end{aligned}$$

We first train ClusterGAN at each client. The central server aggregates local model parameters and updates the ClusterGAN from each client with the global model. Once the ClusterGAN converges during this iterative process, we apply K-Means clustering to the latent space data encoded by the global encoder to identify the cluster (domain) membership for each data sample based on Euclidean distance. As mentioned earlier in this section, ClusterGAN provides a better cluster separation in the latent space than in the original data space. Therefore, we apply K-Means to the latent space data. Since we have no prior knowledge of the number of domains *K*, we adapt an iterative search algorithm from our prior work^41^: We search for the optimum number of clusters (domains) incrementally. Given a specific domain number *NumDomain*, we evaluate the clustering quality by computing the average Silhouette score $score_{avg}$ across all *N* clients. We record the optimal domain number *BestNum* based on the highest average Silhouette score *MaxScore*. If the current score does not improve *MaxScore*, we increase *NumDomain* and proceed to the next round of clustering and cluster quality evaluation. In addition, an early stopping criterion is applied such that the algorithm stops when $score_{avg}$ does not improve for a number of rounds controlled by the variable *Patience*. The process described above is shown in Algorithm 1. After determining the optimal domain number *K*, we apply federated ClusterGAN again to obtain the final domain labels $\mathscr {D}_i$ for $X_i$ in each client.

**Algorithm 1:**
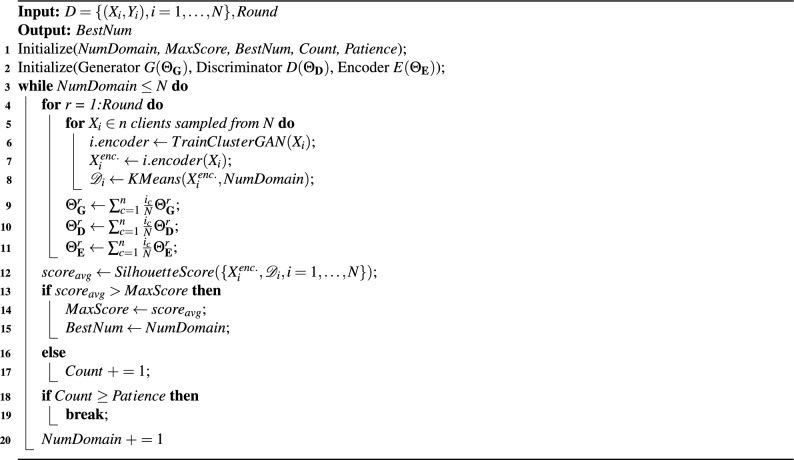
Data-driven Domain Number Estimation

#### Federated multi-domain learning

**Figure 4:**
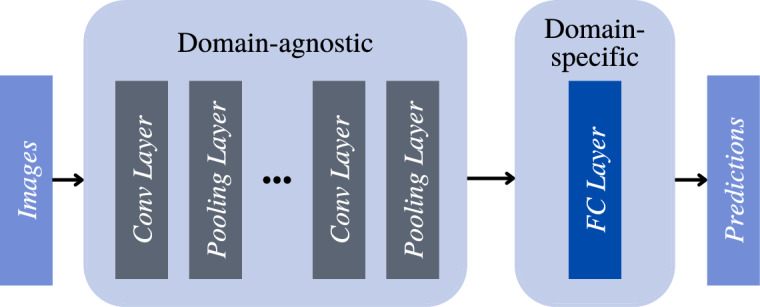
Federated multi-domain learning architecture.

After obtaining the domain labels $\mathscr {D}_i$ for each $X_i$ from federated ClusterGAN, we learn customized classification models for all the domains. A previously reported study indicates that data from different domains can still share a large amount of low and mid-level visual information such that establishing individual domain-specific models will lead to loss of shared information across domains^34^. To preserve the shared information while learning domain-specific knowledge, we apply federated multi-domain learning by developing a Convolutional Neural Network (CNN) *g* that is composed of domain-agnostic layers with a set of parameters $\theta$ for obtaining shared information across domains and domain-specific layers with a set of parameters $\phi$. We define the $n^{th}$ layer of the CNN as the domain-specific layer and the rest of the layers to be domain-agnostic layers. We know from prior research that the end layer of a CNN has lower representation capacity compared to other layers in the architecture and is thus more sensitive to domain-specific information^43^. We, therefore, choose *n* to be the last fully connected layer of the CNN architecture shown in Fig. 4.

#### Graph-based domain knowledge fine-tuning

We obtain customized classification models for data from different domains via federated multi-domain learning. To further fine-tune the domain-specific parameter $\phi$, we adopt a Graph Attention Network (GAT)^44^ to model relationships between domains. We fine-tune $\phi$ by treating each domain-specific $\phi$ as a node in a graph. Due to the intrinsic nature of the graph architecture, a Graph Neural Network (GNN) can effectively learn relationships between the nodes. This makes GNNs an excellent choice for modeling domain relationships. GAT overcomes several limitations of prior GNNs, such as the GCN: it allows different weightings to different neighboring vertices (domains) in the graph, thereby enhancing the interpretability of domain relationships^45^. Moreover, it does not make any assumptions on edge connections between the nodes (domains)^46^, aligning well with our scenario where domain relationships are unknown. Additionally, GAT is computationally efficient^44^.

**Figure 5:**
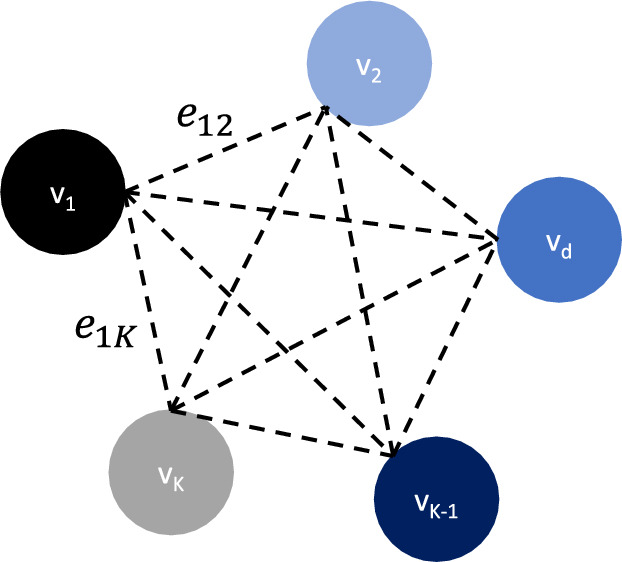
Illustration of GAT architecture. $v_1, v_2, \dots , v_d, \dots , v_{K-1}, v_K$ are the vertices that represent sets of domain-specific parameter $\phi _{d}$ for each domain *d*, and *e* represents the edge in between vertices. For example, $e_12$ is the edge between $v_1$ and $v_2$, and $e_1K$ is the edge between $v_1$ and $v_K$. The number of vertices in the GAT equals the number of domains *K*.

We define the GAT as $h = (\textbf{V}, \textbf{E})$, where $\textbf{V}$ is the set of vertices that represent domains and $\textbf{E}$ is the set of edges that connects domains. As shown in Fig. 5, $\textbf{V} = \{v_{d}, \; v_{d} \in \mathbb {R}^{M}, \; d = 1, \dots , K\}$, where $v_{d}$ is the set of parameters from the domain-specific layer of the classification model that belongs to domain *d*, $M = \left| v_{d} \right|$ represents the number of $v_{d}$, and *K* is the number of vertices (domains). We denote the output of the GAT as $\varvec{\hat{V}} = \{\hat{v}_{d}, \; d = 1, \dots , K\}$. Each output $\hat{v}_{d}$ is a linear combination of its neighboring vertices $\mathscr {N}_d = \{v_j, \; j \in \textbf{V} \setminus d\}$ weighted by normalized self-attention coefficients $e_{dj}$, as presented in Eq. (2)^44^, where $\oplus$ represents the concatenation operation, $\textbf{W}$ is a learnable weight matrix applied to each vertex $v_{d}$, and $\alpha$ is a self-attention mapping that computes self-attention coefficients. We use scaled cosine similarity instead of the conventional dot product to avoid small gradients during model training for the case study^47^.

2 $$\begin{aligned} e_{dj} = \frac{\textrm{exp}(\textrm{LeakyReLU}(\alpha \left[ \textbf{W} v_{d} \oplus \textbf{W} v_{j} \right] ))}{\sum _{k \in \mathscr {N}_{d}} \textrm{exp}(\textrm{LeakyReLU}(\alpha \left[ \textbf{W} v_{d} \oplus \textbf{W} v_{k} \right] ))} \end{aligned}$$

We obtain $\varvec{\hat{V}} = \{\hat{v}_{d}, \; d = 1, \dots , K\}$ based on Eq. (3)^44^, where $\sigma$ is a nonlinear function, *L* is the number of multi-head attention mechanisms utilized to stabilize the learning process, $e_{dj}^{l}$ is the normalized self-attention coefficients computed from the $l^{th}$ attention mechanism, and $W^{l}$ is the corresponding weight matrix.

3 $$\begin{aligned} \hat{v}_{d} = \sigma (\frac{1}{L} \sum _{l=1}^{L}\sum _{j \in \mathscr {N}_{d}} e_{dj}^{l} \textbf{W}^{l} v_{j}) \end{aligned}$$

## Results

### Dataset

In this study, we used the Wearable Stress and Affect Detection (WESAD) dataset^48^. The data was collected from 15 participants using two devices, i.e., a chest-worn device (RespiBAN) and a wrist-worn device (Empatica E4). The RespiBAN measures accelerometer data, electrocardiogram (ECG), electrodermal activities (EDA), electromyogram (EMG), respiratory signal, and temperature at a sampling rate of 700 Hz. Empatica E4 records accelerometer data, blood volume pressure, EDA, and temperature at different sampling rates. All signals were labeled with baseline, stress, and amusement tags^49^. Following the prior work^50^, we utilized the ECG signal sampled from RespiBAN with baseline and stress tags only. Note that we consider each subject with the individual’s data as a client for federated learning with non-i.i.d data due to intrinsic physiological differences between individuals.

### Experimental setup

#### Evaluation metrics

We evaluated our proposed framework using the following criteria for all the experiments:

- Overall accuracy/F1 score: the accuracy/F1 score across all clients in the test dataset.
- Client accuracy/F1 score: the accuracy/F1 score of the individual client in the test dataset.

#### Implementation details

To preprocess the raw ECG signals, we used an R-peak detection algorithm^51^ to detect ECG cycles according to the locations of R peaks and then converted each ECG cycle to an image of size 128 $\times$ 128. Since the WESAD dataset contains valid data from 15 clients, we randomly selected data from 11 clients for training and the rest of the four clients for testing. We fixed the train/test data split for all the experiments.

Following our prior work^41^, we utilized Stochastic Gradient Descent (SGD) as the training optimizer with a weight decay of $10^{-4}$ and a momentum of 0.9. The maximum communication round is 50, with an early stopping criterion of stopping training after having five consecutive epochs without improved accuracy. We implemented the federated clustering module using the model from our prior work^41^. We trained the ClusterGAN at each client with a batch size of 32 and a learning rate of $5 \times 10^{-4}$ for five epochs. We chose the standard ResNet50^52^ as the CNN backbone architecture for multi-domain learning due to its remarkable performance on image classification tasks and training efficiency. We froze domain-agnostic parameters from all convolutional layers of the pre-trained ResNet50 and enabled parameters from its last fully connected layer to be adaptable to each domain. We trained the multi-domain learning module using a batch size of 32 and a learning rate of $10^{-3}$ for 20 epochs. Random search is employed to optimize the hyperparameters. We applied a five-layer GAT model with all layers having $K = 5$ attention heads followed by an Exponential Linear Unit (ELU) activation layer. We applied a dropout rate of 0.2 to avoid overfitting. The GAT was initialized using Xavier initialization^53^ and trained to minimize the cross-entropy loss with a learning rate of $5 \times 10^{-4}$ for five epochs. For all the experiments, we set the seed to 42, repeated each experiment 10 times, and reported the average values.

#### Baselines

To demonstrate the effectiveness of our proposed method, we compare our model with the following two baselines:

- Dynamic-Fusion Federated Learning ($DF\_FL$)^54^: A modified version of the FedAvg algorithm that dynamically selects the participating clients based on local model performance and performs model fusion according to participating clients’ training time. It yields excellent model performance with non-i.i.d health data.
- Cluster-driven Graph Federated Learning (FedCG)^30^: A state-of-the-art federated learning algorithm that addresses intra-client data heterogeneity by utilizing a teacher-student model, cluster-specific models, and a Graph Convolutional Network (GCN) that connects the cluster-specific models.

### Effect of intra-client data heterogeneity

**Figure 6:**
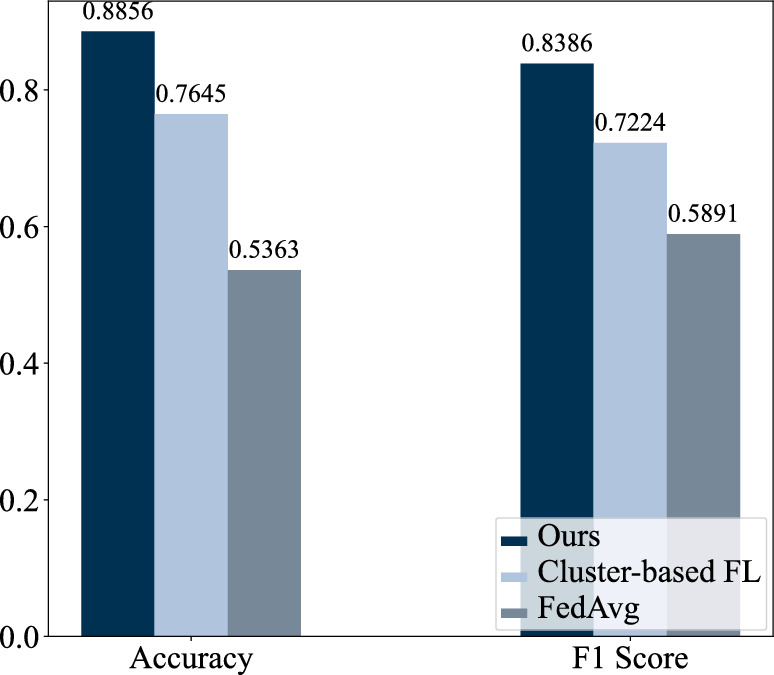
Overall accuracy and F1 score comparison with FedAvg, cluster-based FL, and the proposed algorithm on stress-level prediction.

As described in the Introduction, there exist two types of data heterogeneity, i.e., inter-client and intra-client data heterogeneity. In this section, we demonstrate the effect of data heterogeneity on the client classification model performance by comparing our proposed method with FedAvg^10^ and cluster-based FL. We present the corresponding accuracy and F1 score in Fig. 6. FedAvg aggregates all client models to form a global model and sends back the shared global model to each client without considering inter-client/intra-client data heterogeneity. We observe that FedAvg yields a prediction accuracy of 53.63% and an F1 score of 0.5891, indicating that the global model fails to provide a good generalization across all clients when there exists data heterogeneity.

Cluster-based FL aims at reducing inter-client data heterogeneity by grouping similar patients such that clients within a cluster share similar data distributions and those across different clusters have distinct data distributions. Following our prior work^41^, we performed cluster number estimation, trained federated ClusterGAN, and assigned a cluster id to each client based on the latent embedding from the ClusterGAN. We then applied FedAvg for clients in the same cluster. The cluster-based FL achieves an accuracy of 76.45% and an F1 score of 0.7224, which is approximately 12% lower in accuracy and 0.12 lower in the F1 score compared to our proposed model. The result suggests that considering inter-client data heterogeneity in the algorithm helps improve classification performance. However, the performance is adversely affected by the effect of intra-client data heterogeneity. Our proposed algorithm considers intra-client data heterogeneity and thus significantly improves the accuracy and the F1 score.

Note that identifying outliers, i.e., clients with data distributions significantly diverging from other clients, is beyond the scope of this paper. Prior work on mitigating FL data heterogeneity has not addressed the problem of outliers. This is an area we aim to explore in future research. Additionally, outlier clients in federated learning are often considered malicious attackers, and there exists literature that utilizes anomaly detection techniques to identify and remove such attackers^55–57^. Techniques for detecting outliers to improve the privacy of the FL will be investigated in future work.

### Comparison with baselines

We present the performance comparison between the proposed federated clustered multi-domain learning algorithm and the selected baselines. As shown in Table 1, our proposed approach achieves an overall accuracy of 88.56% and an overall F1 score of 0.8386, outperforming the $DF\_FL$ and FedCG baselines. Since the $DF\_FL$ model does not differentiate heterogeneous data distributions within each client, its mediocre performance demonstrates that the one-size-fits-all federated learning model does not generalize well. The knowledge learned from other clients does not apply to new clients with different data distributions. Our approach is significantly superior since it identifies intra-client heterogeneous data distributions (i.e., domains).

**Table 1:** Overall prediction accuracy and F1 score of the proposed model and baselines.

Method	Overall accuracy (%)	Overall F1 score
$DF\_FL$^54^	48.04	0.6479
FedCG^30^	84.15	0.7776
Proposed method	88.56	0.8386

**Figure 7:**
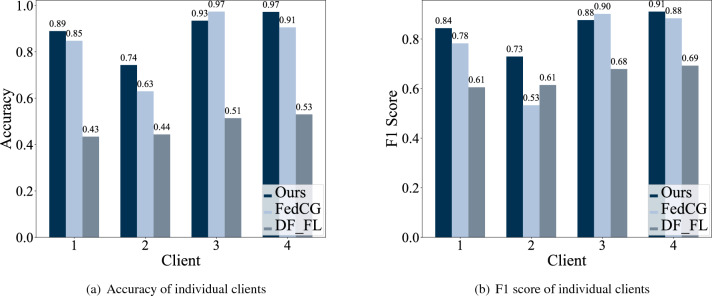
Client accuracy and F1 score on stress-level prediction.

The proposed method obtains around a 4.4% increase in accuracy and an increase of 0.06 in F1 score compared to FedCG. The FedCG utilizes a GCN that defines an adjacency matrix based on pre-determined parameter similarity between vertices/domains. The GAT does not make assumptions about the connections between vertices/domains and assigns different weights to the connecting edges via the attention mechanism^44^. Our results suggest the GAT is a better graph representation learning model with learnable weight coefficients between domains.

We next compare the client accuracy and F1 score obtained from the proposed method and the baselines shown in Fig. 7a and b, correspondingly. The results manifest the robustness of our approach since it outperforms $DF\_FL$ and FedCG on three out of the four clients in the test dataset. The $DF\_FL$ yields low accuracy and F1 score on all clients. This low performance can be attributed to significant inter-client domain differences between clients in the training and test datasets. By comparing the proposed method with FedCG, we observe an increase of at least over 4% in accuracy and an increase of at least 0.03 in the F1 score in Client 1, 2, and 4, while FedCG achieves slightly better accuracy and F1 score in Client 3. Both methods did not perform well in Client 2, indicating that Client 2 may contain different data distributions from the other three clients in the test dataset. To conclude, our model outperforms FedCG in terms of both client accuracy and F1 score.

**Table 2:** Client training time of each module of the proposed method.

Module	Client training time (s)
ClusterGAN	145.4
CNN for multi-domain learning	527.34
GAT	69.68

We further provide the client training time with a breakdown of the time consumed by each module of the proposed method shown in Table 2. Note that we assume all clients to be trained in parallel. We observe that the CNN for multi-domain learning requires a longer training time compared to the ClusterGAN. This difference is due to the longer training epochs of the CNN compared to the ClusterGAN since they have a similar model size.

To better quantify the impact of the number of unknown domains on the overall training time, we express the time complexity of the proposed method as $O(K \cdot T_{NN}(GAN) + K \cdot T_{NN}(CNN) + K \cdot P^2)$, where *K* is the number of unknown domains, $T_{NN}(\cdot )$ represents the time complexity of a given neural network architecture that can be computed based on the work of He and Sun^58^, and *P* is the number of domain-specific parameters. Each term in the expression of the overall time complexity corresponds to the time complexity of each module in the proposed algorithm. The last term is derived based on the work of GAT^44^. We notice that as the number of unknown domains *K* increases, the time complexity of each module of the proposed method will increase, resulting in increased overall training time. Methods that reduce the amount of training time associated with the increase in unknown domain numbers are to be investigated in future work.

**Table 3:** Computation and communication cost comparison with baselines.

Method	Model size (MB)	Computation cost (MFLOPS)	Communication cost (min)
$DF\_FL$^54^	1212	977.47	120
FedCG^30^	175.15	144.72	51.4
Proposed method	Total: 172.88	147.21	50.87
	ClusterGAN: 81.18	68.54	
	CNN for multi-domain learning: 89.67	78.46	
	GAT: 2.03	0.21	

Additionally, to ensure a more comprehensive comparison between the proposed method and the baselines, we determine their model size by computing the number of model parameters (assuming all of them are 32-bit floats), computation cost in terms of Mega floating point operations per second (MFLOPS), and communication cost in terms of latency in minutes; the results are shown in Table 3. Note that we provide a breakdown of the proposed method by each module. Upon observation, we find that the proposed method significantly outperforms $DF\_FL$, featuring both a smaller model size and lower computation and communication costs. The proposed method exhibits a similar model size, computation cost, and communication cost to FedCG, yet it achieves higher accuracy and F1 score. We thus conclude that the improvement in prediction results is attributed to the proposed algorithm itself, rather than other factors, such as increased model size.

### Effect of federated ClusterGAN

In this section, we further demonstrate the effectiveness of our federated ClusterGAN module in identifying domains across all clients. To illustrate the superiority of latent space clustering via ClusterGAN, we replace the federated ClusterGAN module in the proposed algorithm with a teacher-student model^30^, which is effective for domain adaptation, and compare it with the algorithm without replacement. The teacher-student model consists of a teacher network and a student network, where the teacher classifier provides domain pseudo-labels as targets to guide student network training. Note that the work of Caldarola et al.^30^, which minimizes the cross-entropy loss between the domain labels obtained from the teacher and the student model, is inspired by the work of Asano et al^59^. In their research, they proposed an alternating minimization algorithm for self-labeling. We used the same number of clusters as the hyperparameter for a fair comparison. Table 4 presents the accuracy and the F1 score using the ClusterGAN and the teacher-student model. Results show that our federated ClusterGAN outperforms the teacher-student model by 4.34% in accuracy and 0.0293 in F1 score, highlighting the advantage of utilizing the federated ClusterGAN.

**Table 4:** Comparison of overall accuracy and F1 score on federated clustering methods.

Federated Clustering module	Overall Accuracy (%)	Overall F1 score
Teacher-student	84.22	0.8093
ClusterGAN	88.56	0.8386

### Effect of graph attention layer

We next investigate the effectiveness of the auxiliary GAT in the graph-based domain fine-tuning module. To demonstrate that the auxiliary network enhances classification model performance, we conducted an ablation study by removing the GAT. We also compare the GAT with GCN, which has no attention mechanism. We present the overall accuracy and F1 score in Table 5. We observe that the model without a graph neural network yields an overall accuracy of 84.73% and an F1 score of 0.8109, and using a graph-based auxiliary network improves the classification performance. The model with GAT as the auxiliary network outperforms the model with GCN by a 0.67% increase in accuracy and a 0.0066 increase in the F1 score. This increase may be due to the fact that the GAT learns the weights between vertices via the attention mechanism rather than using pre-defined weights as in GCN. Based on the results in Table 5, we conclude that the GAT as an auxiliary domain fine-tuning module improves overall classification performance.

**Table 5:** Comparison of overall accuracy and F1 score on auxiliary domain fine-tuning methods.

Auxiliary domain fine-tuning module	Overall accuracy (%)	Overall F1 score
None	84.73	0.8109
GCN	87.89	0.8320
GAT	88.56	0.8386

## Conclusion

In this work, we have introduced a novel federated clustered multi-domain learning algorithm to overcome intra-client data heterogeneity while preserving privacy. We have also incorporated a graph attention network as an auxiliary domain fine-tuning module to capture the information between domains. We applied our model to the stress-level prediction task using electrocardiogram signals as a case study. The proposed model outperforms selected state-of-the-art methods by over 4% in accuracy and 0.06 in F1 score. However, our approach is still vulnerable to outliers when a client’s domains exhibit significantly different data distributions from other clients. As part of future work, we plan to explore personalized federated learning to develop customized models for each domain of the clients.

## Data Availability

The dataset analyzed during this work is publicly available at https://archive.ics.uci.edu/ml/datasets/WESAD+%28Wearable+Stress+and+Affect+Detection%29.
