# Author Correction: Federated clustered multi-domain learning for health monitoring

**DOI:** 10.1038/s41598-024-53195-w

**Published:** 2024-01-31

**Authors:** Shiyi Jiang, Yuan Li, Farshad Firouzi, Krishnendu Chakrabarty

**Affiliations:** 1 Department of Electrical and Computer Engineering, Duke University, Durham, NC 27708 USA; 2 Division of Natural and Applied Sciences, Duke Kunshan University, Kunshan, 215316 Jiangsu China; 3 School of Electrical, Computer and Energy Engineering, Arizona State University, Tempe, AZ 85281 USA

Correction to: *Scientific Reports* 10.1038/s41598-024-51344-9, published online 09 January 2024

The original version of this Article omitted an affiliation for Yuan Li. The correct affiliations are listed below.

Department of Electrical and Computer Engineering, Duke University, Durham, NC, 27708, USA

Division of Natural and Applied Sciences, Duke Kunshan University, Kunshan, 215316, Jiangsu, China

The original Article has been corrected.

